# Case report: Disitamab vedotin combined with immunotherapy demonstrated excellent efficacy in scrotal Paget’s disease with Her-2 overexpression

**DOI:** 10.3389/fimmu.2024.1349033

**Published:** 2024-06-26

**Authors:** Jia-Ling Wang, Wen-Jun Meng, Nian Hu, Ji-Yan Liu

**Affiliations:** ^1^ Department of Biotherapy, Cancer Center, West China Hospital of Sichuan University, Chengdu, China; ^2^ West China School of Medicine, Sichuan University, Chengdu, China

**Keywords:** extramammary Paget’s disease, human epidermal growth factor receptor 2, disitamab vedotin, scrotal cancer, case report

## Abstract

**Background:**

Extramammary Paget’s disease (EMPD) is a rare epithelial malignancy, and approximately 30%–40% of EMPD patients overexpress human epidermal growth factor receptor 2 (Her-2). Currently, there are no established standard treatments for advanced EMPD while anti–Her-2 therapy is recommended for Her-2–positive cases.

**Case presentation:**

Here, we report a 51-year-old male diagnosed with advanced Her-2–positive EMPD, presenting with numerous lymph node metastases. This patient received disitamab vedotin (an antibody-drug conjugate, targeting Her-2) combined with serplulimab as first-line treatment. After seven cycles of combination therapy, the patient tolerated the treatment well and the lymph node lesions continued to shrink. However, the patient developed immunotherapy-related pneumonia following the eighth treatment. Hormone therapy was administered while all the anti-tumor therapies were halted. After the pneumonia improved, the patient underwent positron emission tomography-computed tomography, revealing a complete response to his tumor. To consolidate the effect, he received another five cycles of disitamab vedotin monotherapy as maintenance therapy, without experiencing any adverse events. To date, the patient has remained in good health without any recurrence 10 months after drug discontinuance.

**Conclusion:**

Disitamab vedotin combined with immunotherapy demonstrated a long-term clinical benefit in advanced Her-2–positive EMPD. For rare solid tumors with Her-2 overexpression, disitamab vedotin combined with immunotherapy might offer a viable therapeutic choice.

## 1 Introduction

Extramammary Paget’s disease (EMPD) is a rare epithelial malignancy, primarily occurring in the skin with rich apocrine glands, including vulva, scrotum, penis, and axilla (1). EMPD typically presents with asymmetrical white and red scaly plaques, pruritus, pain, bleeding, ulceration, and swelling, mimicking inflammatory skin diseases, thus often leading to misdiagnosis or delayed diagnosis (2, 3). Wide local resection remains the standard treatment for local lesions; nevertheless, 30%–60% of patients with such resection could experience recurrence (1), requiring re-excision or further therapy. Additionally, a tiny proportion of EMPD patients progress to be invasive and develop local or distant metastasis, resulting in a dismal overall survival of 9.8 months in the absence of treatment (4). At present, there have not been established treatments for metastatic EMPD, and treatment experience is based on case reports or small case series. Chemotherapy regimens, such as docetaxel, fluoropyrimidines, S-1, platins, and their combination therapy, are attempted and achieve some progress. Docetaxel is preferably applied in first-line treatment for advanced EMPD patients and the progression-free survival (PFS) is 7.1 months in a multicenter study. However, 100% of patients (*n* = 13) develop grade 3 or 4 myelosuppression (5). Other chemotherapy regimens achieve similar treatment efficacy with PFS of 3.7–9.7 months (6–9). In general, the treatment efficacy is limited with the risk of adverse effects. Therefore, it is essential to find more effective and tolerable treatment options for advanced EMPD. Noteworthily, about 30%–40% of EMPDs overexpress the human epidermal growth factor receptor 2 (Her-2) protein. The driving effect of Her-2 on the oncogenesis of EMPD remains unclear; however, targeted drugs designed for Her-2 could be an essential treatment strategy for advanced EMPDs with Her-2–positive features (10). Data from a multicenter study showed that patients receiving anti–Her-2 treatment achieved a higher objective response rate (ORR) than chemotherapy (100% vs. 45.5%), suggesting the considerable therapeutic potential of Her-2–targeted drugs in advanced EMPDs (7).

Disitamab vedotin (also called RC48) is an antibody-drug conjugate, consisting of monomethyl auristatin E (MMAE, a cytotoxic agent) coupled with hertuzumab (a monoclonal antibody targeting Her-2) via a cleavable linker (11). Attributed to this structure, disitamab vedotin demonstrates a powerful cytotoxicity and, meanwhile, ensures the high stability and targeting *in vivo*. In 2021, disitamab vedotin was first granted for advanced or metastatic gastric cancer patients with Her 2+ or 3+, who had received at least two prior standard treatments (12). Then, multiple clinical trials verified the efficacy of disitamab vedotin in Her-2–positive patients in various tumors, including urothelial cancer, breast cancer, and biliary tract cancer, and so forth (12)., thus indicating a potential treatment strategy for those tumors with high Her-2 expression. Here, we reported a rare case who was diagnosed with Paget’s disease of scrotum and complicated with lymph node metastasis. The patient was treated with disitamab vedotin combined with immunotherapy, achieving a long-time disease-free survival. This might provide potential management for Paget’s disease as well as rare solid tumors with Her-2 overexpressing.

## 2 Case presentation

**Figure 1:**
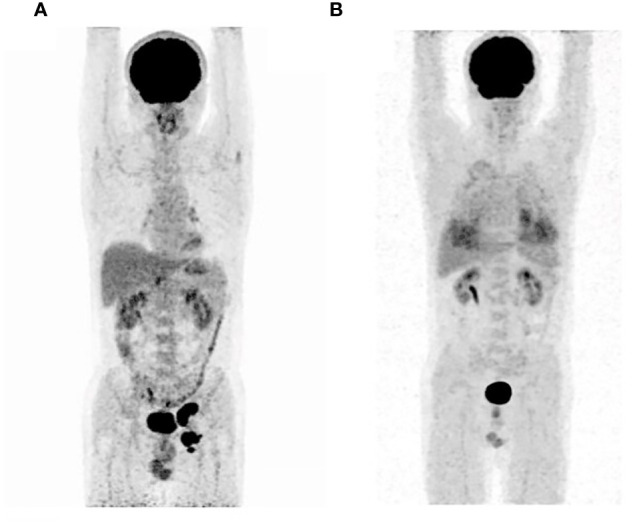
The 18F-fluorodeoxyglucose positron emission tomography-computed tomography before initial treatment **(A)** and after eight cycles of treatments **(B)**.

**Figure 2:**
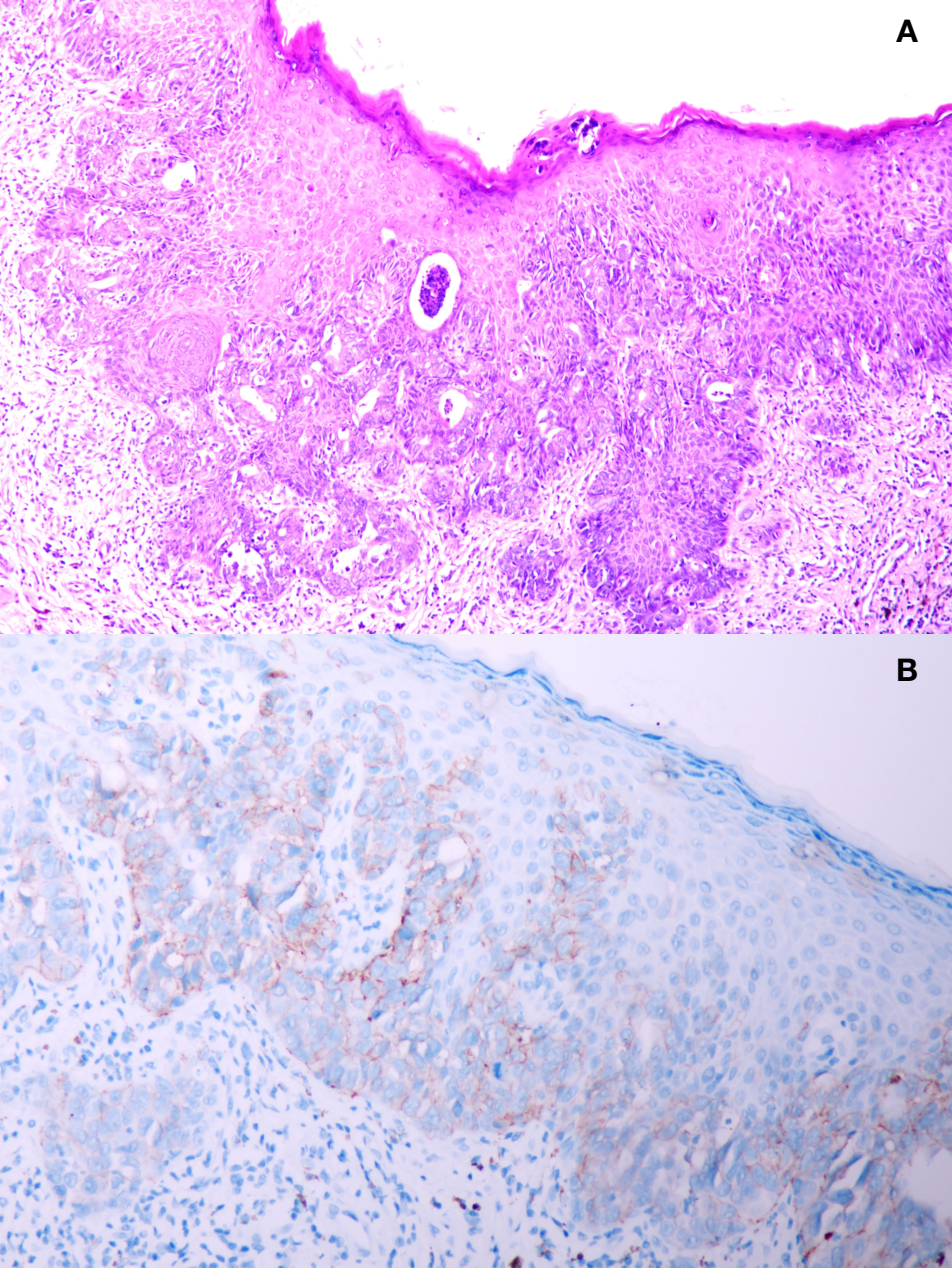
**(A)** Pathology result from scrotal biopsy of this patient; **(B)** immunohistochemistry staining of human epidermal growth factor receptor 2 protein from scrotal biopsy.

A 51-year-old male presented to the outpatient unit due to his scrotal Paget’s disease. Eight years ago, the patient noticed a rash on his left scrotum with redness and fluid, without other symptoms such as itching, pain, bleeding, and so forth. Over the past 8 years, he was treated as “scrotal eczema” with topical medications and his symptoms had not improved. One month prior to his admission, the patient presented with a palpable mass of more than 3 cm on the left groin. The mass did not improve or alter after the anti-infective treatment with amoxicillin. The 18F-fluorodeoxyglucose positron emission tomography-computed tomography (PET/CT) scan from the local hospital showed multiple enlarged inguinal and iliac lymph nodes, suggesting metastasis of malignant neoplasm (**Figure 1A**). On June 8, 2022, the patient underwent left inguinal lymph node dissection and left scrotal biopsy at a local hospital and was diagnosed with EMPD with lymph node metastasis based on postoperative pathology. For further treatment, the patient visited our hospital. The results of pathology consultation were as follows: cytokeratin-7 (+), GATA-3 (+), SOX 10 (−), S-100 (−), MART-1 (−), P63 (−), and Her-2 (++), which confirmed the diagnosis of EMPD (**Figure 2**). No fluorescence in situ hybridization (FISH) was performed to further detect Her-2. The CT scan revealed multiple metastases of lymph nodes, indicating the impossibility of surgery. A comprehensive gene detection was also conducted to guide tumor treatment, but no other significant mutated genes were found.

**Figure 3:**
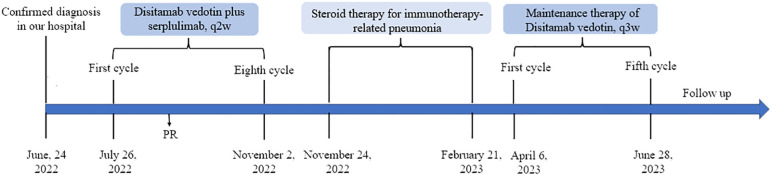
Timeline of the major clinical events from diagnosis to the last follow-up.

**Figure 4:**
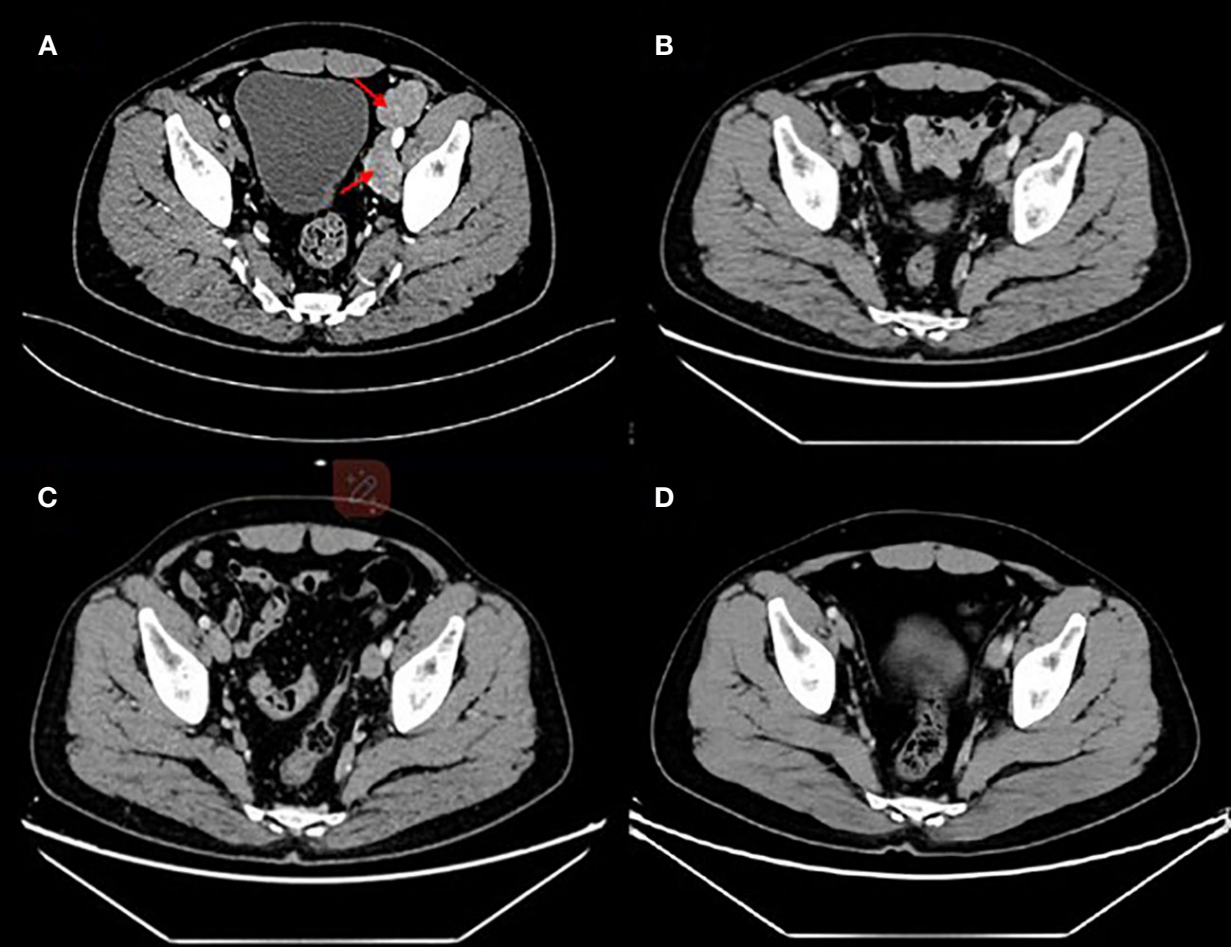
Enhanced computed tomography for efficacy evaluation **(A)** before treatment on 29 June 2022, **(B)** after two cycles of disitamab vedotin plus serplulimab on 7 September 2022, **(C)** after five cycles of disitamab vedotin maintenance therapy on 27 July 2023, and **(D)** 9 months after stopping any treatments on 24 March 2024.

Given that there have not been established criteria for advanced EMPD and the patient refused to accept chemotherapy regimens due to adverse events, we recommended anti–Her-2 drug combined immunotherapy as first-line treatment and the regimens gained the patient’s informed consent (**Supplementary Material**). Before the drug initiation, we informed the patients of the risks of immunotherapy and made a series of conventional safety tests before each medication. On 26 July 2022, this patient started with disitamab vedotin 120 mg plus serplulimab 200 mg every 2 weeks (**Figure 3**). Through seven cycles of treatment, the patient tolerated the treatment well and no adverse events occurred. During this period, enhanced CTs were performed to evaluate the efficacy and results showed that lymph node metastases shrank (**Figure 4**).

On the third day after the eighth infusion, the patient complained of a cough and gradually developed chest tightness and shortness of breath. Chest CT scan showed multiple ground glass, solid shadow, and strip shadow in both lungs. The C-reactive protein was 20.4 mg/L and the highest level of white blood cells was 16.41*10^9/L. Combined with the clinical symptoms and laboratory examinations, this patient was diagnosed with immunotherapy-related pneumonia. A large dose of methylprednisolone sodium succinate was immediately applied, while all anti-tumor treatments were discontinued. After a month of hospitalization, the patient’s symptoms disappeared and hormone tapering therapy was initiated upon discharge from the hospital.

On 21 February 2023, this patient underwent a PET/CT scan and the results revealed that there were no tumor lesions in his body (**Figure 1B**). To prevent the relapse of his tumor, this patient received another five cycles of disitamab vedotin monotherapy as maintenance treatment. During the period of maintenance treatment, the patient tolerated well and no adverse events occurred. Currently, the patient has stopped his treatment for 10 months without any recurrence and maintained a good quality of life.

## 3 Discussion

This report describes a rare case with Her-2–amplified scrotal Paget’s disease with multiple lymph node metastases. The patient received disitamab vedotin as first-line therapy and achieved a long-term clinical benefit. To our knowledge, this is the first EMPD patient who was treated with disitamab vedotin, which could provide a reference for the treatment of rare Her-2–positive solid tumors.

Lymph node invasion is an essential risk factor for the prognosis of EMPD patients and available drugs are quite limited. In a retrospective analysis, data revealed that the 5-year survival rate was 100% for sentinel lymph node-negative patients but was 24% in sentinel lymph node-positive cases (13). For this patient in our report, multiple lymph node metastases were present at diagnosis, and complete resection was not possible. To prolong the patient’s survival, aggressive anti-tumor therapy was extremely necessary.

Her-2 protein is a transmembrane tyrosine kinase receptor and its primary role is to participate in the regulation of cell growth, differentiation, migration, and survival (14). In various cancers, Her-2 has become a unique prognosis factor and an essential therapy target. For EPMD, Her-2 overexpression is thought to be related to the invasion of the disease and lymph node metastasis (15). Nevertheless, that also provides a therapeutic option. Drawing on the experience of treating breast cancer with Her-2 overexpression, trastuzumab or its combination therapy has been employed to treat Her-2–positive EPMD. Trastuzumab possesses an excellent curative effect on cancer cells; however, the killing effect on carcinoma cells is not adequate. For this reason, the combined use of trastuzumab and chemotherapy is often required to enhance the therapeutic efficacy. Previous studies have verified the superiority of trastuzumab with chemotherapy compared with chemotherapy in gastric cancer, breast cancer, uterine serous cancer, and so forth (16–18). Furthermore, trastuzumab combined with systematic chemotherapy in EMPD achieved good therapeutic effects with a median PFS of 13.3 months, better than chemotherapy alone (7).

At present, there has not been established consensus for the first-line treatment of EMPD due to the rarity of this disease. Although his tumor was Her-2 positive, this patient refused trastuzumab plus chemotherapy or chemotherapy due to the potential adverse effects. Taking all these considerations, we recommended disitamab vedotin as a treatment option considering outstanding clinical efficacy in Her-2–positive solid tumors, and the patient chose this strategy. Disitamab vedotin contains hertuzumab and MMAE coupled by chemical bonds. Hertuzumab could help transmit the cytotoxic agents to tumor cells for precise killing (19). Meanwhile, the coupled structure also facilitates the cytotoxic drug to release until reaching the target tumor tissue, minimizing systemic toxicity like conventional chemotherapy. Compared with trastuzumab, hertuzumab demonstrated a more outstanding affinity for Her-2 and exhibited an enhanced ADCC activity ***in*** *-* ***vitro*** experiments (20). Moreover, despite the failure of trastuzumab, disitamab vedotin could still demonstrate outstanding efficacy, which highlights the expanding use of disitamab vedotin (21, 22). In the trials of RC48-C005 and RC48-C009, disitamab vedotin achieved a median ORR of 50.5% in Her-2 positive locally advanced or metastatic urothelial carcinoma patients, 86% of whom experienced failure from previous therapies (23). In a phase 2 trial, disitamab vedotin showed promising clinical benefit and PFS was 4.1 months for Her-2 overexpressing gastric or gastroesophageal junction cancer patients with at least two lines of previous treatment (22). In other advanced solid tumors with Her-2 overexpression, antitumor activity from disitamab vedotin was also observed and 25% of patients achieved ORR. Interestingly, disitamab vedotin still exhibited efficacy in Her-2 negative or unstable cancers, and the ORR was 38% (24). This could be explained that MMAE reaches the tumors guided by hertuzumab and induce a bystander effect to kill neighboring cells (25). Therefore, although the patient did not take the FISH test, we decided to have an attempt about disitamab vedotin in EMPD with Her-2 overexpression given the promising efficacy of disitamab vedotin in Her-2–positive solid tumor. Additionally, the results from the combination therapy of disitamab vedotin are also noteworthy. Preliminary data about disitamab vedotin combined with toripalimab displayed a satisfactory clinical benefit, with ORR of 100% in Her-2 (3+), 77.8% in Her-2 (2+), 66.7% in Her-2 (1+), and 50% in HER2 negative patients with urothelial carcinoma, respectively (26). In a real-world study, the combination of disitamab vedotin with immunotherapy demonstrated similar results with an overall ORR of 88.9% and manageable safety (27). These results suggested that immunotherapy could provide a synergistic effect for the application of disitamab vedotin; therefore, we incorporated immunotherapy to enhance the anti-tumor efficacy. Although immunotherapy had to be withdrawn due to adverse events, disitamab vedotin still could guarantee tumor regression. Currently, multiple clinical trials focusing on disitamab vedotin are ongoing, which will provide valuable information on its effectiveness and help determine its potential as a treatment option for cancer patients.

## 4 Conclusion

In summary, we reported that a Her-2–positive EMPD patient with multiple lymph node metastases achieved a CR after the first-line treatment of disitamab vedotin and serplulimab. Additionally, the patient had a good tolerance for disitamab vedotin. This was the first EMPD case that received disitamab vedotin and that demonstrated a long-term clinical benefit. Although further clinical study should be implemented to prove the efficacy, disitamab vedotin showed an excellent prospect in advanced rare solid tumors with Her-2 overexpression.

## Data availability statement

The raw data supporting the conclusions of this article will be made available by the authors, without undue reservation.

## Ethics statement

The studies involving humans were approved by Institutional Review Board of West China Hospital, Sichuan University. The studies were conducted in accordance with the local legislation and institutional requirements. The participants provided their written informed consent to participate in this study. Written informed consent was obtained from the individual(s) for the publication of any potentially identifiable images or data included in this article.

## Author contributions

J-LW: Investigation, Writing – original draft. W-JM: Conceptualization, Writing – original draft. NH: Writing – original draft, Investigation. J-YL: Conceptualization, Supervision, Writing – review & editing.
