# Supplementary material for: Case report: Disitamab vedotin combined with immunotherapy demonstrated excellent efficacy in scrotal Paget’s disease with Her-2 overexpression
Source: Front Immunol. 2024 Jun 26;15:1349033. doi: 10.3389/fimmu.2024.1349033 (PMC11233530; doi:10.3389/fimmu.2024.1349033)
Supplement: Supplementary file 1 [file DataSheet_1.zip › Informed consents of the patient.pdf]

四川大学华西医院

医患沟通表

科室：特需医疗中心·全科医学科医疗单元 **登记号：0034118357**

姓　　名：伏果　　性　　别：男　　年　　龄：50岁

床　　号：B823床　　入院时间：2022-07-21 14:30

目前诊断： 1.阴囊Paget病伴腹股沟淋巴结、盆腔肿块转移；2.肝囊肿

沟通地点： B823床旁

沟通时间： 2022年07月25日 17:08:19

沟通参与人员： 院方：刘继彦 李玉函医疗组

患方：患者及家属

沟通事由： 告知病情及治疗相关

沟通内容： 患者目前诊断如上，患者为晚期肿瘤病人，胸腹部增强CT提示：左侧髂窝盆腹膜外间隙、左侧腹股沟区多发软组织结节及肿块，大者约4.1×2.9cm，呈明显、均匀强化，考虑肿瘤性病变，淋巴源性肿瘤可能性大。肝多发稍低密度结节，弱强化，大者约1.4cm，转移瘤？肝多发囊肿。双肾小囊肿。前列腺钙化灶。腹主动脉旁多发小淋巴结显示。十二指肠小憩室。右侧髂骨、耻骨结节状、片状稍高密度影，请结合临床及骨扫描。双肺小结节，直径约0.2-0.4cm，请随诊。双肺下叶散在炎症。心脏未见增大。患者目前考虑广泛淋巴结转移及肝转移可能，患者疾病属于罕见病，没有标准治疗方案，提供以下两种治疗方案：1.常规化疗±靶向药；2.新型ADC药物+免疫治疗。患者病理提示：〈左侧阴囊〉乳腺外Paget病，Her-2中等强度不完整膜（+）。患者考虑选择Her-2（+）的新型ADC药物+免疫治疗。已向患者交代这些药物可能无效，可能存在不良反应，特此告知。

沟通结果：（确认）病情，（选择）ADC药物+免疫治疗。

医师签名： 刘继彦 李玉函　　**患方签名：** 伏果

日　　期：2022年07月25日　　日　　期：2022.7.25

四川大学华西医院

临床研究知情同意书

科室：特需医疗中心·全科医学科医疗单元 **登记号：0034118357**

患者姓名：伏果 性别：男 年龄：50岁 床号： B823床

尊敬的病员朋友：

您好！我们拟邀请您参加临床医学研究。请您仔细阅读本知情同意书的具体内容，如有不清楚的问题或术语，可以与医务人员进行咨询和讨论，以便您决定是否参加临床医学研究。

为了开展临床医学研究，促进生物医学进步，提高疾病诊断、治疗水平及人民健康水平，希望您能授权我院在充分保护您的个人基本信息安全及隐私的前提下，保存和利用来源于您在我院此次就诊期间，包括门诊、住院、急诊、随访等就诊时的医疗资料，如病历、影像学资料、检验检查数据等以及临床诊疗过程中剩余的样本（即在满足常规医学检查、病理诊断及治疗后的剩余样本：包括手术切除组织、活检组织和细胞、粪便、血液、尿液、胸水、腹水、痰液等），用于今后的临床医学研究。

本研究所收集与分析的资料我们将严格保密，未经您的同意，绝不向任何个人或第三方泄露

您的个人基本信息和隐私信息。

您参加本研究完全是自愿的，您可以在任何时间退出本研究。本次研究已通过四川大学华西

医院生物医学伦理审查委员会审查同意。

感谢您的参与，祝身体健康！

一、研究目的与基本情况介绍

我们将在国家卫健委等行政管理部门的监督和指导下，按照现行法律、法规、规章和规范性

文件，如《人类遗传资源管理条例》《涉及人的生物医学研究伦理审查办法》《中华人民共和国

个人信息保护法》等规定，妥善长期保存和利用您的医疗资料及临床诊疗的剩余样本。相应的医

疗资料及临床剩余样本将仅用于医学研究，或保存于“四川大学华西医院生物样本库”（以下简称“样本库”）用于医学研究，但不涉及人体基因方面的医学研究。

二、风险和注意事项

1. 我们将依法保存您在此次就诊期间或以后就诊时的医疗资料和数据信息。

2. 我们保存您的生物样本是在常规诊疗过程（检验、检查、病理诊断、治疗等）后的剩余样

本，不会因今后研究目的额外采集。

3. 您的诊疗剩余样本保存与研究，不需您承担任何费用。

4. 本机构使用您的医疗资料以及剩余样本开展临床医学研究时会对您的身份数据信息进行脱

敏并保护。

三、本项目的获益

您的参与将会推动医学技术进步，从而可能获得更有效的疾病诊断、治疗方法，这将惠及您

以及与您相似疾病的其他病员朋友。您可能无法从签署本《知情同意书》获得经济补偿和诊疗费用减免等直接利益。

四、个人隐私权保护

我院将严格遵守《中华人民共和国个人信息保护法》和《中华人民共和国数据安全法》，以

及其他隐私和个人信息保护的法律法规，并采取下列保密措施：

1. 建立受试者信息安全管理制度及个人信息保护制度，对所有保存的样本采取去标识化管理；

2. 安全保存样本和数据，对所有样本和/或数据设置访问权限；

3. 当生物样本或制备的研究材料提供（以科研为目的的样本转移和流通）给其他机构及其研

究者时，任何研究机构或个人都不会获得您的个人隐私信息；

4. 研究所得数据可能公开发表或公布，但会对您的姓名及其可辨识您身份的相关信息进行保

护；

5. 您完全自主决定是否同意签署本“知情同意书”，即使签署后您也有权在任何时间、无需任何条件决定退出，也不会因为您的任何决定受到任何歧视和报复，您的医疗待遇和合法权益都会得到保障。

6. 您的研究资料将保存在四川大学华西医院，研究者、研究主管部门、伦理委员会及法律法规、政策允许的单位和个人可以查阅您的医疗记录。

五、其他事项

为公众健康、国家安全和社会公共利益，国家可以依法使用和共享我院收集的生物样本。国家也可以根据抢险救灾、疫情防控或者其它需要下达指令性任务，我院将遵照相关法律法规履行相应责任。

患方知情选择：

- 医务人员已向我充分解释和说明了本知情同意书的目的、风险、操作过程、担责和自愿原则等以上内容，并回答了我关于本《知情同意书》的所有相关问题。
- 我理解参加本活动完全是自愿的并有随时无条件退出的权利，我的任何医疗服务与权益不会受到任何影响。
- 我知道，签署《泛知情同意书》，并不意味可以免去我的常规诊疗费用。

我已经认真阅读并理解上述内容，经过慎重考虑，我明确 同意 （"同意"或"不同意"）授权四川大学华西医院在充分保护我的隐私和个人基本信息安全的前提下，采集、保藏、利用我此次就诊期间或以后（包括门诊、住院、急诊、随访等）就诊时的医疗资料（包括病历、影像学资料、各类检验检查数据等）和临床剩余样本（例如手术切除物、活检组织和细胞、粪便、血液、尿液、胸水、腹水、痰液等）用于临床医学研究；同意因科研的需要，将去除个人身份标识化后的医疗资料以及临床诊疗过程中剩余的样本交由其他机构及其研究者进行临床医学研究；我已知晓并理解，虽然在研究中可能发现有关我的重大健康问题，若未发现或未告知并不构成四川大学华西医院和相关研究机构及人员必须履行的义务，四川大学华西医院和相关研究机构及人员不必为此承担相关法律责任。

患者签名：[illegible]　　签名日期：2022.7.25

患者的联系电话：18981604523　　联系方式：

与患者关系：　　联系方式：

医务人员陈述：

我已明确具体地向患者说明了本项目的情况，特别是参加本项目可能产生的风险与受益、免费与补偿、自愿与保密等伦理原则和要求，并回答了患方所提出的所有问题。

医护人员签名：[illegible]　　联系方式：18980601768　　签名日期：2022.7.25

姓名：伏果　　性别：男　　年龄：50岁　　床号：B 823床

疾病介绍及治疗建议：

1. 患者目前诊断：1. 阴囊Paget病伴腹股沟淋巴结、盆腔肿块转移；2. 肝囊肿。根据患者目前的病情，有免疫靶向治疗的指征，需使用维迪西妥单抗联合斯鲁利单抗进行免疫靶向治疗，未见免疫靶向治疗禁忌症。

2. 替代医疗方案：化疗±靶向。

注意事项及治疗风险：

免疫靶向治疗有治疗疾病的作用，但同时存在严重的毒副作用，并且因患者个体差异及某些不可预料的因素，可能在治疗中或治疗后发生一些并发症或风险，造成患者身体不同程度的损害，严重者可能致患者死亡。为使患者及家属充分知情并自愿选择，现将有关免疫靶向治疗相关并发症及风险告知如下（有些不常见的并发症及风险可能没有在此列出）：

1. 一般毒性：头晕、乏力、食欲减退、恶心、呕吐、咳嗽、发热、脱发、体重下降、电解质紊乱、外周性水肿、肌肉骨骼痛、药物过敏反应及输液反应等。

2. 特殊性器官毒性：

（1）皮肤毒性：皮疹、瘙痒、皮肤干燥、指甲异常、痤疮及脓疱性皮疹、皮肤毛细血管增生症，过敏反应如血管性水肿和荨麻疹，极罕见中毒性表皮坏死松解症和多形红斑等；

（2）内分泌毒性：甲状腺功能减退、甲状腺功能亢进、垂体炎、原发性肾上腺功能减退、高血糖等；

（3）胃肠毒性：腹痛、腹泻、结肠炎等；

（4）骨骼肌肉：类风湿关节炎、肌炎、肌痛、关节炎、关节痛等；

（5）输注反应：发热、低血压、呼吸困难、血管性水肿、心动过速等；

（6）神经毒性：重症肌无力、格林-巴利综合征、无菌性脑膜炎、脑炎、脊髓炎等；

（7）血液毒性：自身免疫性溶血性贫血、再生障碍性贫血、血小板减少、获得性血友病等；

（8）眼毒性：葡萄膜炎、巩膜炎等；

（9）高血压，甚至难以控制的高血压，脑血管意外等，严重者导致死亡；

（10）脏器溃疡形成、出血、穿孔、梗阻等；

（11）肝脏毒性：肝功能损害、肝功能衰竭、肝纤维化等；

（12）肾脏毒性：肾功能损害、肾功能衰竭等；

（13）感染甚至引起脓毒血症，严重者导致死亡；

（14）静脉血栓及动脉栓塞，甚至肺栓塞，心脑血管意外等危及生命。

（15）肿瘤超进展，即肿瘤在免疫治疗后短期内快速长大；

（16）免疫靶向治疗相关性皮炎、肺炎、心肌炎、胃肠炎、肝炎、肾炎及内分泌疾病等，严重者导致死亡。

（17）肿瘤不能控制或复发。

（18）其他难以预料或无法防范的不良后果。

3. 上述不良反应和风险，情况严重者需要较长时间治疗，甚至终身服药，少数患者可能致残疾、死亡。

4. 除上述不良反应和风险外，拟行的免疫治疗方案主要不良反应和风险还包含相应药品说明书所载内容。患方在治疗前有权并应当查看相关药品说明书（特别是不良反应部分），患方有权要求医师就存疑或不能理解部分说明书内容进行解释说明。

5. 肿瘤免疫治疗药物预估费用，具体以费用清单载明的金额为准，只有部分药物在指定情况下才能报账，详细情况请咨询医院医保管理部门或者参保地医保行政部门。

患者知情选择：

● 医务人员已经明确具体地告知我替代医疗方案、治疗中/治疗后可能发生的并发症和风险，并且解答了我提出的与该治疗及本知情同意书内容有关的问题。

● 我同意在治疗中医师可以根据我的病情对预定的治疗方式做出调整。

● 我理解我的治疗可能需要多位医务人员共同进行。

● 我并未得到免疫靶向治疗百分之百成功、安全的许诺。

● 一旦发生前述风险，为保障生命安全，我授权医师根据情况决定我的抢救诊疗方案，并同意支付所发生的全部诊疗费用。

● 我知道在本次治疗开始之前，我可以随时签署拒绝该治疗的意见，以取消本知情同意书的决定。

我已经认真阅读并理解上述内容，经过慎重考虑，我明确 同意 （“同意”

或“拒绝”）使用维迪西妥单抗联合斯鲁利单抗进行肿瘤免疫靶向治疗，并愿意承担该选择可能导致的风险。

患者签名：伏[illegible]　　　　签名日期：2022.7.25

若患者不能或者不宜签字，由患者的近亲属或者委托代理人在此签字确认：

患者近亲属或委托代理人签名　　　　与患者关系：　　　　签名日期：

医务人员陈述：

我已经明确具体地告知患方替代医疗方案、治疗中/治疗后可能发生的并发症和风险，并且解答了患方关于该治疗及本知情同意书内容的问题。

医务人员签名：[illegible]　　　　签名日期：2022.7.26
